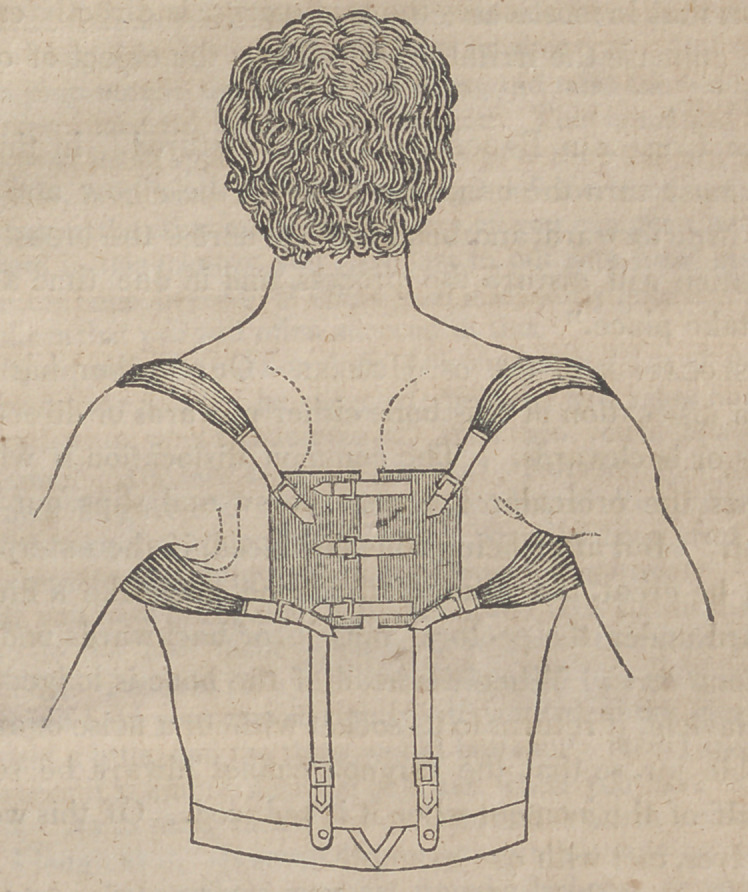# Lectures on Anatomy, Surgery, and Pathology; Including Observations on the Nature and Treatment of Local Diseases

**Published:** 1829-01

**Authors:** 


### REVIEWS.

---

ART. III.—*Lectures on Anatomy, Surgery, and Pathology; including observations on the nature and treatment of Local Diseases: Delivered at St. Bartholomew's Hospital.* By JOHN ABERTETHY, F. R. S. Surgeon to St. Bartholomew's and Christ's Hospitals, &c. 2 vols. pp. 748. Boston, 1828.

(CONCLUDED.)

We resume the analysis of this interesting work.

Lecture 28th, the first of the 2d volume, begins with accidents of the shoulder joint; which from the frequency of their occurrence, not less than the importance of the parts affected, deserve the gravest consideration. Admitting, moreover, of little delay, there is no time to send to a distance for one who may have made surgery a special object of attention, and every physician, therefore, should be prepared to treat them on the most approved principles.

FRACTURED CLAVICLE. No bone is more easily reduced: none more difficult to retain in its proper place. Those who have used the roller of Desault, must have found its application attended with difficulties, which, in reference to its retention, are still greater. The bandage of Boyer has seemed to us, on the whole, more simple and efficacious; but even this cannot always, in private practice, where patients take great locomotive liberties and are often impatient under every kind of compression, be easily kept adjusted. Disappointed in their expectations, and fatigued by the reapplications that generally become necessary, many practitioners have dispensed with both; and contented themselves with merely placing the arm in a short sling, a method which in fact answers very well, except in females where *all* deformity should of course, as far as possible, be prevented.

The bandage preferred by Mr. A. and used in St. Bartholomew's, appears (for he describes it loosely) to be the same with that employed in Guy's hospital, and figured by Sir Astley Cooper, in his work on Dislocations, and on Fractures of the Joints. Its application may be seen in the following plate copied from Sir Astley's valuable treatise.

The clavicle is liable to Dislocation at both its extremities, but the sternal is far oftener displaced than the scapular. The treatment is the same as when the bone is fractured.

Mr. A. here takes occasion to advise his pupils to examine "accidents with gentleness:" an admonition which may with great propriety be extended to the practitioners of this country. We have often been shocked at witnessing the unfeeling energy, with which surgeons prosecute their examinations of parts in a lacerated or morbidly sensible state. They act as though the *ultimate relief* ought to reconcile their patients to the harshest manipulations. These, however, they had better reserve for the dead subject; where they cannot excite inflammation, nor aggravate that spasmodic action of the muscles, which so often constitutes the main difficulty to be overcome. We know of no duties in the discharge of which, it is more commendable to conjoin the *suaviter in modo* with the *fortiter in re* than those of our profession, for the plain reason, that in most cases the said *suaviter* is directly calculated to appease the irritation, which it is the object of our art to subdue.

The Coracoid Process may be fractured. In this case "you must turn the hand round, bring the elbow and shoulder a little forward, and bend the arm across the breast; then no motion will disturb the process, and in due time a union will take place."

Dislocation of the os Humeri. Our author has never seen a dislocation of this bone either upwards or directly forwards or backwards. The common dislocation is where it "bursts the orbicular ligament below and slips out of the socket." But after being thus displaced, if the external violence be great, the head of the bone may take a direction forward under the pectoral muscle, or backwards under the *latissimus dorsi.* When the head of the bone is lodged under the clavicle, it returns to its socket without a noise or any perceptible jar, so that the surgeon cannot always be sensible himself of the moment when it is reduced. Of this we have ourselves, met with one example.

In attempting to reduce an ordinary dislocation of this bone, it is of the utmost importance to take the muscles off their guard. The patient's attention should be diverted from them and occupied on some other subject. It is the irritable state of the muscles that opposes the reduction.

"I have, says Mr. A. reduced several dislocations myself, by a sort of legerdemain. I should, in that case, say to a patient, I do not know what is the matter with your arm; but you hold it so tightly I cannot move it; cannot you leave your elbow to me? I will not hurt it; let me move it. Oh! now you are holding it. Does the motion hurt you? No, it cannot do that. Well then, when you have got the patient to let you do this, you have only to engage his mind with something or other—and there is nothing more likely to engage his mind, than to talk to him about the way in which he met with the accident,—and then you have only to push it in. Now if you do this, you make a lever of the bone, applying the prop at the same time.

I stated that a dislocated jaw was to be reduced on this principle; but I said I would not at that time explain it. If you move a patient's elbow upward and downward, backward and forward, even to a considerable extent, without putting any prop near to the head of the bone, what do you do? Seeming to do much you do nothing at all; the bone does not move one jot from its situation. But suppose I put a prop near to the head of the bone, and raise the head of the bone, then I raise the head into the socket. This is making a lever of the bone itself, applying the prop as near to the head to be raised as possible, in order to increase the length of the lever in applying the power to the other end. Well, I say, if you can get a person off his guard in this manner, you have just to put your hand up to the head of the bone, depress the elbow, and it instantly goes in. But do not believe that you will often succeed in this.

Well, suppose you cannot elude their vigilance, (that of the muscles,) and I say it is not be expected; nobody, who knew how alert the muscles are, would undertake it. Well then you must overcome their power. And how are you to do this? Not forcibly—your conduct must be governed by certain known laws. You must be acquainted with the action of muscles. Muscles have great power, but no muscle can act in a moderate degree unremittingly.

Well, you have to engage the attention, and then you have to pull; yet you do not pull them violently, or in a way to create alarm or opposition. I should always be inclined to say to a patient, 'Upon my life you hold your arm so tightly—if you would but give way—only yield a little, and the thing would be done.' Now I speak of a case where it is simple, near the cup, and where you have simply to draw it. As to using tackle and pulleys, and so on, in a case of this kind, I laugh at it. It seems like breaking a butterfly on a wheel. You have only to make a man fast round a bed-post, or something of that sort, put a napkin round him and pull; pull so as you will not soon be weary of pulling. If you set persons to pull in a violent manner they get tired, and the patient gets tired. Then they tug, then they slack a little. Then they pull, then they slack a little again; and in this way they never will succeed. Oh! it is such a regular pull-baker pull-devil concern, it is quite shocking."—pp. 10—12.

When the head of the bone is driven under the pectoral muscles and lies nearly in contact with the clavicle—

"I never then would attempt to undertake to reduce it without having pulleys, tackles, and every sort of machinery at hand, such as should ensure my success. And the way to do it is this:—You must have a cord, surrounded with tow, and afterwards sewed up in leather or cloth; loop it sufficiently to slip over the shoulder, bring it against the inferior costa of the scapula, and bind the scapula up. In short, you make a sort of pad, such as people wear who carry pails on their heads. Then you have straps coming from the pad, which should be fixed in the wall with four screws; but let the straps be very far apart, otherwise you would squeeze the patient's body; and let them be fixed so as not to impede the head of the bone coming into its socket. Then by pulling at the elbow you will bring it into its socket. The best way is to put some buff leather round the elbow, and I have always been in the habit of doing it, by making a half hitch, or clove hitch as the sailors call it. When this is put round over the buff leather, one of the ends will draw and the other will not. The one will wedge or jam, and you draw the other as tight as you please. What does it signify if you should stop the circulation? It does not signify for the moment. You put on two half hitches, and pull the bone down to, or under the socket, towards the inferior costa of the scapula, and take great care that the extension be unremitting. Now, under these circumstances, as soon as the muscles are engaged in resistance, you say to the patient, 'Oh, sir, you are holding—you are opposing, there is nothing that will hurt you; do give way, do yield a little;' and as the muscles become weary, you find the head of the bone to come further and further from under the pectoral muscle; and, if you are using pullies, you say to the patient, 'Just half an inch more, sir; just half an inch more.' Then, when it is below the socket, good may be done by putting a prop under the head of the bone and depressing the elbow. You may, with your own hands, use this expedient for reducing the bone. I never failed but in one case."—p. 13.

Dislocations may be reduced after a considerable time. The adhesion of the torn ligaments to the surrounding parts, is what opposes their reduction. If upon applying a moderate force for some time the head of the bone is not found to move, all further attempts should be given up. As to bleeding, the warm bath, and other relaxing measures, Mr. A. condemns them, as unnecessary to the abatement of muscular contraction, and inappropriate to the dissolution of the new adhesions.

On compound dislocations and on fractures of the os humeri, on fractures of its neck, and on fractures of the scapula, our author has a single page, in which we see nothing particularly worthy of being extracted.

The succeeding lecture is on DISLOCATIONS and FRACTURES of the ARM and HAND, including the FINGERS. A dislocation of the elbow inwards, is a rare occurrence. The most frequent dislocation of this joint, is that which drives the radius off its tubercle and plants the ulna in its place. A luxation of the elbow joint is to be ascertained by observing the relative position of the olecranon process and the condyles, especially the internal. The fore arm is generally bent to a half right angle, and you can neither flex or extend it. To reduce it—

"You are to pull the bones downward and inward, for the purpose of making extension, and, by using the force steadily, they slip into their proper places. And you are assured the dislocation is reduced, from this circumstance, that you can bend and extend the fore-arm to its utmost limits, and turn the radius into a state of pronation and supination.

But you may reduce the ulna and not the radius; and the radius may be dislocated without the ulna. In either case, the reduction of the radius is to be effected by a separate operation. You reduce this by making a lever of the bone, applying a prop to the part, and bending the fore-arm. You press down the head of the radius. I now state to you the principle. You make a lever of the bone, raising the one end and depressing the other; and, in short, if you know the principle, why, you can apply it to a vast number of cases.

The radius may be dislocated separately, either forward upon the os brachii, or backward; and when it is dislocated backward, why it is a difficult dislocation to reduce. To reduce it, you have to pull it steadily at a half right angle, and to press back the os brachii at the same time. That is the mode of reducing it, as far as I am capable of judging; but it certainly is a difficult dislocation to reduce." —p. 18.

When the fore arm is dislocated backwards, the coronoid process is often fractured, and the arm is permanently extended. As our author is silent respecting the mode in which this dislocation should be reduced, we shall transcribe from Sir Astley Cooper, the methods which he has been accustomed to employ.

"This dislocation is easily reduced by the following means: The patient is made to sit down upon a chair, and the surgeon places his knee on the inner side of the elbow-joint, in the bend of the arm, and taking hold of the patient's wrist, he bends the arm; at the same time he presses on the radius and ulna with his knee, so as to separate them from the os humeri, and thus the coranoid process is thrown from the posterior fossa of the humerus; and whilst this pressure is supported by the knee, the arm is to be forcibly but slowly bent, and the reduction is soon effected. It may be also accomplished by placing the arm around the post of a bed, and by forcibly bending it while it is thus confined. I have also reduced the limb by making the patient, whilst placed upon an elbow chair, put his arm through the opening in its back, and then having bent the arm, the body and limb being thus well fixed, the reduction of the dislocation was easily effected."—*Treatise on Dis. and Frac. of the Joints*, p. 334.

In treating compound luxations of this joint, after replacing the bones, close up the wound with sticking plaster, and then varnish over the dressings with a varnish made by dissolving sealing wax in alcohol; keep the limb steady, and sponge it perpetually with water, so as to keep the heat down; and the patient will generally do well.

On fractures of the lower end of the os humeri, of the olecranon process, and of the fore arm, we shall extract nothing.

Accidents to the WRISTS. The wrist may be dislocated outwards or inwards, when the lateral ligament must be ruptured, or it may be thrown backwards or forwards. After reduction, which is easily effected—

"Treat it as a fractured fore-arm, that is, by putting a little tray of pasteboard, which will support the hand in a continued line with the bones of the fore-arm, not letting it drop down by its own weight, and steadying it by lateral splints, or at any rate by a splint put into the palm of the hand, not to suffer the hand to turn round.

But SPRAINED WRISTS are perpetually accidents of tedious cure; and for a very good reason; because the injuries are not inflicted on the ligaments or joints only, but all the sinews are inflamed; inflammation takes place—there is a gumminess, and swollen state of the parts, which render the hand crippled for a considerable time. Now, in this said position of the fore-arm, all the sinews run in a straight line; there is no twisting of the fore-arm, and all the sinews run in a straight line, when the fore-arm is in that situation. Let it move about, and the sinews are twisted; and if the sinews are inflamed, the twisting and motion very materially aggravate the inflammation. In short, in a common sprained wrist, if there was no ulceration or inflammation, the wisest thing a surgeon could do, would be to support it with a splint, keep the hand steady, and then keep down the inflammation as you would in a case of dislocation of the *olecranon*."—p. 22.

Accidents to the CARPAL and METACARPAL BONES. Mr. A. never saw a dislocation of any one of the carpal bones. But the bones of the metacarpus may be luxated. In the treatment of this affection our author disapproves of Mr. Hey's recommendation, to saw off the end of the luxated bone, for it opens the joint, and exposes the synovial membrane. He would at least wait until all the inflammation had subsided, and see if the patient was likely to suffer great inconvenience. It would then be time enough to proceed to the operation. When the bones of the carpus are shattered, as by the bursting of a gun, Mr. A would replace as many of them as possible, put the hand in a case to keep it steady, sponge the part continually, and attend to the diet and habit of body of the patient.

In dislocations of the fingers, that of the last joint only presents any difficulty. It cannot be reduced by extension, for there is no part to get hold of. Mr. A. learned how to manage such a case from one of his pupils, Mr. Curwarden. "He said, instead of trying to extend it, you had better try to bend it a little more, and that will humour the flexor tendor, and by only getting it so far bent that the proximel end of the phalanx is raised up you will have it in."

Lecture 30 embraces, in its first part, accidents occurring about the HIP JOINT the largest and in many respects the most important articulation of the body. Contrary to what might be expected from the anatomy of that joint, a dislocation upward and backward, has often happened in Mr. A's. practice. The round head of the bone generally turns towards the sacrum, and can be felt on the dorsum of the ilium. The limb is shortened, and the toes turned in. Sometimes the bone remains in that position, but in other cases it sinks down into the sacral notch. In both instances the process of reduction is the same.

"You have nothing to do but to pull the knee with the thigh bent at a half right angle upon the pelvis, then the head of the bone will come down below the acetabulum, and having got it there, it generally slips into the socket. You cannot pull it over the acetabulum; you cannot pull it over the great ridge that is there, but you bring it down where the bone has no great rise in it, for there is below the acetabulum a groove, between the acetabulum and the tuberosity of the *ischium,* in which the *obturator* moves, and it is along this groove, provided you pull at the angle specified, the bone will come, and when it gets below the brim of the acetabulum, it slips in."—p. 28.

The dislocation, occasionally, is inwards, when the toes are turned in the opposite direction. The method of reduction is the same.

When the bone is dislocated downwards, its head is lodged in the obturator foramen, the limb is elongated, and the toes are generally inverted:

"Then what is to be done in this dislocation? Why, to be sure, a man who sets himself to pull at the leg, would be pulling it further from the socket. You would perhaps say, pushing would be the best thing to be done; but you cannot push it over the brim of the acetabulum. Well, this can only be reduced by making a lever of the dislocated bone, applying a prop at the head of it, and a power at the other end; and thus wou,d you lift it into the socket."—p. 30.

It may happen that the dislocation is directly upwards, when the thigh is shortened and cannot be bent:

"Well, then, you are to pull it down into the socket, you are to extend it, and if you depress the head of the thigh bone, making a sort of lever of it, that would be a very considerable auxiliary in the reduction. However, it may be brought down by simply pulling; but a little pressure at the one end, with a sort of lever at the other, would very considerably tend to assist it."—p. 31.

Fractures of the long neck of this bone may be confounded with its dislocations, except that in the obturator foramen, from which they may always be distinguished by the shortening of the limb.

"Sometimes the bend and neck of the thigh bone split, and the thigh bone gets wedged in between them. The cases are therefore often complicated. That splitting of the neck of the thigh bone is not at all uncommon; but when the fracture is on the outside of the *articular ligament,* the bone may crack, and be simply cracked, where the neck of the thigh bone is joined on to the body of the bone, and there may be very little suppuration between the parts, but the patient is unable to go one step forward. I have heard of cases where they have walked, but then it must have been cases, I am sure, of the neck of the thigh bone having been wedged into the body of the bone, not where it is simply cracked, because where it is simply cracked, I am sure they would never bear on their legs to get one step forward."—p. 31.

"I would treat the fracture of the *neck* of the thigh bone as I would treat a fracture in any other part of the bone, and lay them on their side. If a person can be fairly laid on the side, and a splint put on the bone, so that the weight is supported by its own gravitation, then that will do. The rule is, that every longitudinal inch should press equally on the splint, so that you should be able to take up the limb, aud carry it about on the splint as if upon a tray. Well, you have to put a counter splint. In the fracture of the neck of the thigh bone, where there is a counter splint, why the pressure of the pelvis on the head of the thigh bone keeps the bone steady, and causes it to press against the part which is broken. But all half measures are bad. I do assure you, I tell you no lie when I tell you that I have attended many cases where there have been fracture of the neck of the thigh bone, and where people have gone about without the possibility of knowing that ever there had been such a fracture; but all half measures are bad. You must therefore turn them fairly upon the side. And how are they able to go to sleep without moving? Why, by lying fairly on their side, the gravitation fixes them in that position; attend to the points of the pelvis, and I am sure they will never move. For my own part, I have no objection to people lying on a soft feather bed. All the trouble I have with the patients is for the first dozen hours, in watching them that they do not move; and if they are laid on a feather bed, they get sunk and fixed in that feather bed in such a way, as that out of it they cannot move afterwards. Well, that is all I wish you to attend to on that point."—p. 34.

Mr. A. has known the neck of the thigh bone fractured after it was dislocated upon the dorsum of the ilium. This is a bad case. On removing the extending power, the limb is immediately shortened; and again great decripitude is the almost inevitable result. In such a case we should be disposed to employ extension for many weeks, keeping the upper end of the bone as near as possible, beneath the acetabulum. Some species of artificial joint would perhaps be thus formed.

Diseases of the Hip joint. Inflammation of a common kind may establish itself in the hip joint, and eventuate in suppuration. Such a case is often confounded with lumbar abscess; but may be distinguished from that malady by the limp of the patient, and the soreness which he feels on pressure about the acetabulum. The treatment consists in perfect rest, low diet, successive doses of Calomel, leeching, and, after the soreness has abated, the application of a blister. Many cases of this kind call, however, for general bloodletting. With respect to rest, it is all important, and when the patient cannot otherwise be induced to keep the limb still, a splint should be so applied, to prevent all motion in the joint. Of blisters, it may be observed, that they are often applied so early in the disease, as to do harm instead of good. In the event of a profuse discharge of matter, on opening an abscess of this kind, it is necessary to administer Bark and Opium, as in all similar cases.

So much for idiopathic inflammation of the joint. But it is often diseased in consequence of an unhealthy state of the constitution. In such cases rest is not so indispensible, and the patient should be allowed to walk about on crutches that his general health may be improved; he should not, however, bear weight upon the affected limb. In these cases, leeches and blisters generally do harm. The disordered state of the constitution must be corrected by appropriate means, and the joint simply fomented with tepid water.

But the hip joint is liable to a much more formidable disease, denominated in some of the books *ischias*. It is of a scrophulous nature, and commences in the ends of the bone or in the acetabulum, or both. The bony parts become light and spongy, they ulcerate, and the head and neck of the os femoris are progressively absorbed; suppuration often happens round the joint; and, finally, in many cases, anchylosis takes place. This is a "horrible disease," and does not, in general, terminate, so that the patient can walk, under three years. On the subject of its treatment our author observes:

"Now I am convinced that a great deal of good may be done, and the good to be done is in keeping the part quiet, repressing all inflammatory action, instituting counter-irritation, and taking care that the counter-irritation does not disturb the health of the patient. And if ever there should be a fit of inflammatory action coming on, the patient should immediately be laid in bed, the peas taken out again, or beads, or whatever you may have put in to cause the irritation; and nothing but soothing treatment encouraged. You must not produce irritable action, while the internal disease is inflammatory.

To suppose that all cases will do well, is to suppose an absurdity. To suppose that those cases will do well in a hospital, is to suppose an absurdity; for the air will not admit of it, with regard to the patient's general health. But many of such cases will do well."—pp. 46–47.

When anchylosis is about to take place, it is of great importance to keep the limb extended, and the toes directed forward. When it is flexed upon the pelvis, the patient is of course subjected to great inconvenience. It was in this case that our intrepid countryman, Dr. Rhea Barton, projected and successfully executed the operation of sawing through the neck of the bone, so as to form an artificial joint; a full account of which was published in our journal of last year. Of this operation, which does honor to American Surgery, Mr. Abernethy says nothing.

Dislocations of the Knee joints, being comparatively simple in their treatment, we shall pass by, with a single extract on the necessity of keeping the limb extended:

"As in the knee, so in the *elbow-joint*. The great object is to keep them straight. In either of these joints, where the motion is likely to be imperfect after the injury heals, it is most important to keep the limb extended."—p. 48.

In this sentence there is either an oversight or a misprint. No Surgeon would advise the elbow joint to be kept straight, when anchylosis was about to occur. This would, indeed, be in express violation of our author's own instructions. Thus at p. 19 he observes:

"In all accidents occurring about the *elbow-joint*, where it is likely to be attended with some inconvenience or defect afterwards, it is a great objecct to have the fore-arm bent, because, if it is not, the fore-arm remains extended, and he cannot apply the hand to any part of his person, nor can he even feed himself; so that one would attempt to reduce it after a considerable period of time."—

In injuries of the knee joint, the condyles are liable to be fractured obliquely, and even twisted out of place. These are vexatious cases. It is difficult either to restore or preserve the part in its proper situation. The leg must be extended, kept still and the inflammation kept down.

In treating FRACTURES of the LEG, Mr. A. inclines to the practice of Pott, or the half bent position of the limb; though in oblique fractures he would, in imitation of Hunter, keep the limb extended.

"You will know when you have set the patient's leg right, by attending to the *spine* of the tibia and the *skin;* for granting it was wrong, if it was laid in a horizontal situation, you would ascertain it by tracing the spine; if in a contrary direction, by tracing the skin. Now, you may laugh at all this, and it would be a most unimportant remark indeed, if it did not happen that a bandy-legged man broke his leg. If he broke his leg, and you were to put it straight, you will just put it wrong. I remember a bandy-legged man once in that situation; notwithstanding all the pain the surgeon was putting him to, he smiled and said, 'Sir, I think you are putting my leg straight, and, if you are, that is what I never remember its being before!'"—p. 49.

We have heard an anecdote still more in point. It was told, if we are not mistaken, by the venerable Dr. Worthington, of the District of Columbia. When a young physician, the Doctor, in a country excursion, stopped at a tavern, where a "Dutch Doctor" was engaged in setting the leg of a "bandy shinned" countryman. When the apparatus was applied, and the leg made *quite straight*, the self complaisant and self taught Surgeon, exclaimed—*Daer! it pe'sh petter dan it wash pefore.*

ACCIDENTS about the ANKLE JOINT are of frequent occurrence and deserve most attentive consideration. Few injuries are more common than A SPRAINED ANKLE, in which the ligaments are stretched, and sooner or later, may become inflamed, and the inflammation may involve all the surrounding soft parts. In many cases however, it happens with this joint, as with the knee and hip, that no inflammation comes on, and the parts remain weak and relaxed. This is the fault of the constitution, which must be corrected, and strengthened, before the injured parts will recover. The cure is always slow, and sometimes is never perfect, a feeling of weakness remaining throughout the patients life. In such cases it is improper, or, at least useless, to make many applications to the affected part. Cooling washes, leeches, and blisters all do harm. Rest, friction with the hand, and a roller carried from the lower extremity of the limb upwards, are all that can be done to the part affected with advantage. In persons of a feeble constitution, and nervous temperament, these accidents are often followed by a great aggravation of those morbid states, and we have met with few conditions of the system more unmanageable. Even when the constitution is healthy, a sprain is not followed by immediate inflammation. The parts are stunned and weakened, and, under such circumstances, the practice of applying cold or evaporating washes, is extremely ungrateful to the patient, and actually injurious. *Hot* water is then, one of the best applications, or hot stimulating lotions. The limb should be kept on a level with the pelvis; and, if the pulse be weak, or the patient chilly, he should take a draught of hot spirit and water, or a dose of laudanum. When inflammation supervenes, and the heat begins to rise, the parts should be perpetually bathed with tepid, or cool, but not cold water, and the most perfect rest injoined.

But to return to our author. The most common DISLOCATION of the ankle, is that which presents the foot turned outwards, the tibia thrown inwards from the astragalus and os-calcis, and the weight of the body made to rest upon the fibula. The interosseous ligament is sometimes torn, and the fibula often fractured, about three inches from its lower end. The integuments round the outer ankle are often pinched between the bones, while those of the inner ankle are lacerated, constituting a compound dislocation. These are most serious accidents, we mean the last, and the custom once was to resort to immediate amputation. Against this practice, our author enters an energetic protest. The parts should be replaced, care being taken to disengage the integuments from between the bones, the wound should be closed with sticking plaster, and then varnished over, the limb should be raised, and the joint kept constantly sponged. General bloodletting will not often in Mr. A's. opinion be proper. The inflammation generally subsides in four or five days; but sometimes large abscesses form about the joint, which should be opened as soon as a fluctuation is perceived, that the pus may not travel along the tendons and under the fasciæ. If the patient is not likely to do well, amputation may be resorted to at any time.

It often happens, after injuries of the ankle joint, that the weight of the body does not fall over the centre of the plantar arch, but inwardly; and in some persons, there is a certain degree of congenital malformation of this kind in both ankles. It causes the knees to approach each other, and such persons are called "knock-kneed." Much may be done to remedy this deformity, by the patient himself. The inner quarter of his shoes ought to be made high and stiff; in walking and standing, he should not turn out his toes; and in placing his foot, should bring down its outer edge as firmly as possible. Mr. A. is of opinion that irons are rarely of any benefit in these cases.

Of other dislocations of the ankle joint, and injuries of the foot, our author says but little, and that little we shall not analyse; but conclude this branch of the subject with a reference to the ISCHURIA, which, according to his experience, is a frequent consequence of injuries of the foot and leg. In this affection, it is improper, in his opinion, to resort to the Catheter, which by its irritation is apt to keep up the suppression. It is only necessary to administer a mild cathartic and apply leeches to the perineum, when the urine will pass freely.

We now leave the skeleton and its diseases, for the muscles, chiefly of the trunk of thebody, to which Mr. A. has devoted the greater part of his 31st, 32d and 33d lectures. His descriptions are not given, but the reader is presented with many edifying and amusing remarks, on the relations between Myology and Surgery. Without attempting to analyse that which is already in a state of disintegration, we cannot resist the inclination to transcribe a few specimens.

"Now, a knowledge of this opening (the abdominal ring) I hold to be a very important point. Ruptures are often taking place there; and what would you think of a surgeon who would take hold of a rupture in this place, and who would use all his might to push it through the very walls of the belly, into the belly itself. Then it is important to know how to find that aperture; and you may always know that, if you recollect these anatomical facts. I will venture to say, there is no subject so fat, that you cannot distinguish the angles of the pubes, and the jutting parts to be found there; then, knowing that, by poking your finger along the bones, near to the angle, push it a little upwards, and then it goes into the the abdominal ring. Let the skin be off or on, and I do the same thing with my finger. Now here I begin to say, what I shall often repeat, I dare say—that it is really necessary, as far as I am capable of determining, for gentlemen to come to London, or some large school, where they may have opportunities of dissecting and understanding the structure of the body. But there is a great deal of anatomy, and the very best part of anatomy, for *practical* purposes, that you may always remember without a subject, by recollecting that you continually carry about with you a subject in your own persons; and I hope you will all put your fingers into your abdominal rings, and learn for yourselves. And if you reduce a hernia by piecemeal, poking it in, bit by bit, in this aperture, then that is the mode of proceeding."—pp. 63-64.

"Then, as to wry necks in general, they are the result of the irregular action of muscles; and many muscles are concerned in the production of the wry neck. Now in those cases of wry neck which result from the irregular action of muscles, if the sterno-cleido-mastoideus be chiefly affected, what is to be done? Why, you must endeavour to tranquilize the muscles. There are many of those cases entirely the result of a disturbed state of nerves, as I believe, caused by disordered digestive organs. And whether this be universally the case or not, I will tell you of one absolute undoubted instance of it. This happened in a tall boy, at school. He was seized with a wry neck. They leeched him, and blistered him, and made bad worse, and, after a week, he was sent up to town. I say he was a tall, lank boy; and upon my life, I thought a *pullet's* neck could not have been more twisted round than his was; that, you know, admits of a particular turn from the mechanism of the vertebræ of the neck, in order to let the fowl put its head under its wing to sleep. But so much was this boy's neck twisted, that I told him to lie on a sofa, or couch, or bed, and to support the head with pillows; never to sit upright; never to put the head in a position to demand muscular action for support, since, if the muscles did act, they would act in this faulty manner. I told him to foment his neck by flannels; to keep it in a kind of tepid bath; to keep himself in a kind of perspiration; and I applied the whole of my attention to put his digestive organs to rights. His bowels were all wrong—his tongue furred, and of a bad colour. A week elapsed, and his stomach and bowels got into a decent state, and his wry neck was entirely gone. But you see if this is neglected in the outset, then muscles get a habit of perverse action, and you have wry necks established beyond removal, even when the digestives are put to rights, though that I believe to be a most direct and efficient mode of cure in every instance. But I know that there are cases of wry neck, where people have their necks twisted, and the muscles put into such an irritable state of action, that the cases do not yield quickly, even to what I should consider the most judicious and appropriate treatment. But, now, in reading books of surgery, you find the proposition of dividing the sterno-cleido-mastoideus muscle, in order to set people's heads right on their shoulders. Now, for a very great part of my life, I did not know what those people meant by this. I never could meet with a case where such an operation could for a moment have been rationally thought of. Then, as curious cases are something like misfortunes, that is, they never come alone, in one year I met with *four* cases requiring an operation in the sterno-cleido-mastoideus. All these cases appeared to me to be of the same nature. I mean to say, it appeared to me that the muscle was originally mal-formed; that the clavicular part of the muscle was shorter than it should have been. It was irritable, anddrew the mastoid process towards the collar-bone. And note the consequence. The people—children they were, for they were not more than fifteen or sixteen years of age—had all grown awry in their backs. The vertebral column had become completely deformed. And it is natural to suppose that that would be the case, for if any thing oblige a man's head to lie pretty much in contact with his shoulder, he is under the necessity of bending the vertebræ of the loins to the opposite side, in order to support the gravity of his head perpendicularly upon the *sacrum*. The inversion of one vertebræ, creates the necessity for another inversion. I made a longitudinal incision in the sternal portion of the muscle, cut it fairly through, and up started the head perpendicularly upon the neck. I did not touch the clavicular portion of the muscle at all; that did not seem to be at all faulty, and when the clavicular portion was set right, all seemed right. And I have great satisfaction in informing you that these children became straight in their backs. Now that is a very curious thing, that the curvatures of their spines became involved without any mechanism. In one of the children, indeed, a second contraction took place after about a year and a half. I shall not stop now to explain the process—that is explained in the surgical Lectures—but I divided it a second time, resolved that the child should grow up to its full stature without deformity of the vertebral column. I have seen several cases since; and there was a student here who chose to have the operation done on his neck; not that it was in any considerable degree deformed. He would have done very well for a *courtier* in the court of Alexander the Great, where it was the fashion, in imitation of their monarch, to walk with their heads a little twisted. But however, he chose to have it done, and done it was, and with a very satisfactory result to him. He then could do with his head what he could not have done before."—pp. 76–78.

"*Deltoid Muscle.*—If there is any part that surgeons should be acquainted with, it is the gap between the deltoid, and the clavicular portion of the pectoral muscles. I put my finger into that gap in my own person, and I stop the beating of the pulse at the wrist. Suppose a man has got a bleeding in his arm, which there is a difficulty in stopping, how are you, as a surgeon, to stop it? By passing your finger over the first and second ribs, pressing the artery, and you stop it at once. When I talk about this sort of thing, and hear a medical man say he cannot do it, I always say, there are others that can. And I tell this story of a lady who was examined by a great number of learned doctors. They could not tell what was the matter with her; they had never met with such an intermittent pulse in the whole course of their lives, nor any thing at all to equal it, and they pronounced their opinion that she could not live a moment, and left the room; and all this time she was tweedling with this artery at the shoulder."—pp. 82–83.

"*Orbicularis Palpebrarum.*—The tendon of the orbicularis palpebrarum is very important; the knowledge of its situation is what gives you the power of opening the *lachrymal bag* with the greatest facility. The knowledge of its situation is what gives you the power of performing the operation for the *fistula lachrymalis* without the slightest difficulty. You put a knife below the orbicular palpebrarum, keep it within the ridge of the orbit, and where does it go? Into the lachrymal groove, and you carry it on till you can carry it no further, for it is resisted by bone. You divide the bag, put the probe down into the nose, clear away any obstruction that may be there, and you will find that your probe rests upon the nasal process of the superior maxillary bone."—pp. 89–90.

"Now I will just say, with respect to *system*, that there is a great deal in that. In acquiring a knowledge of any science, system is almost indispensably necessary; but is especially so in the medical profession. With this view, the abdomen is divided into regions; and from want of attending to this, I have known something of this nature occur elsewhere. The examiner has asked a young gentleman who presented himself for examination, 'What do you see upon opening the abdomen?' The chap has stared, and looked aghast, and stuttered out, 'wh—wh—wh—what—what do I see, sir?' 'Ay?' 'Why, th—there's—there's the liver, sir.' 'Well?' 'An—an—and there's the stomach, sir. An—an—an—and—and there's—there's the spleen, Sir. And there's the cœcum, sir.' Now, then, just let me tell you, that you will do well to proceed systematically and to pack up your knowledge as you go along, in such order, that when you are asked for it, you will at once know where to find it."—p. 83.

The latter part of lecture 33, is on the Anatomy and Physiology of the EYE, in which we find nothing particularly deserving of notice: we shall, however, present to our readers the closing paragraph.

"And now I have done. I have told you of all the circumstances —of all the artifices which Nature has employed for lubricating the front of the eye-ball, and keeping it continually transparent. In no other part of the body have you such a series of contrivances, the uses of which you so well understand. Every thing seems required, and every thing required is met with, and every thing met with seems to be admirably adapted to its function; so that I cannot but think that the lachrymal parts of the eye may serve as an instance of the effects of a Designing Cause, operating in the formation of Nature's works."—p. 109.

We come now to the viscera. Lecture 34, opens with the Anatomy and Physiology of the STOMACH. It presents nothing new; and, except the last paragraph, nothing which deserves to be transferred to our pages. Of the reality of what it sets forth, few persons can entertain a doubt.

"For my own part, I can see no permanent source of strength but in digestion. You may give *bark;* ay, and if the stomach will digest it, it will give strength, but I know not how it will give it otherwise. We ought to caution patients, not to take too much exercise after eating, but to keep themselves quiet, and not to allow their minds to be disturbed, for you know the eighth pair of nerves come from the brain to the stomach, and if the mind be agitated and disturbed, will the stomach digest food, or will it even receive it? No; there is a great sympathy between the mind and the stomach. If a man has received some intelligence which really distressed or annoyed him—thoroughly distressed him; if a merchant heard of the loss of a great adventure at sea; if a surgeon heard that a patient of his had died in whose fate he had taken great interest, who he thought would have recovered, and by whose recovery he would have had credit secured to himself; or, if a lover heard that his mistress had cheated him just at the time he was sitting down to dinner, would he eat his dinner do you think? Oh! no; or if he heard of the tidings just after he had dined, would what he had taken digest? Oh! no. Well, to secrete, there must be vital actions regulated by the nervous system; and, in every part of the physical body you will see that fact proved. Now I go on. The food is digested, but what does it become! Not chyle in the stomach, but a kind of pulpy adhesive sort of matter—sticky sort of matter, of no very pleasant odor. People vomit after they digest their food, when they have had a blow on the head; and what are we to call that? It is generally called chyme. I may inform you, that there have been opinions entertained, that the right end of the stomach is better calculated for solid food, and the left for the fluid. But these are things, I believe, concerning which we have no absolute knowledge; and, therefore, we are ignorant of them."—pp. 114–115.

Then follows an account of the intestines, in which likewise, we discover nothing novel.

Having spoken of the alimentary canal in its healthy state, our author proceeds to treat of its Morbid Anatomy: under three distinct heads,—Læsions of the *peritoneal coat* of the *mucous lining* and of the *intermediate tunic.*

*Peritoneal coat.* This part is the seat of inflammation, both acute and chronic. The latter may endure for a long time, and gradually occasion two kinds of effusion—serous and fibrinous—one constituting ascites, the other cementing the organs to one another.

"The *viscera* are all united together; sometimes there is no cavity at all; sometimes in trying to separate the viscera I have torn the bowels asunder without separating them; the peritoneum every where rough and thick, parts adhering, and the vacancies between those parts filled with fluid and tubercles, the peritoneum studded with tubercles."—p. 122.

*Mucous coat.* This membrane, is liable to ulceration, and the ulcers often eat through the other tunics; but it rarely happens that an aperture is formed, so that the contents of the tube can escape, for when the inflammation extends to the peritoneal covering, adhesion of the part to a contiguous intestine, or to the walls of the abdomen, is apt to take place. Ulceration may occur in the stomach, the great intestines and the small intestines, but it is most frequent in the last.

*Intermediate parts.* These are subject to a scirrhus induration and thickening, the other coats remaining entire. Ulceration sometimes takes place. The pylorus is especially liable to such affections. The alimentary canal, like all the other mucous canals, is liable to strictures; some of which are cancerous, others not. They may occur in any part, but are most common in the *rectum,* which in all cases of obstinate constipation, should be examined with the finger. All parts of the canal may experience intus-susception, from irregular and spasmodic action, and large portions are sometimes separated by mortification, and discharged. The extent of involved bowel, is now and then very great. A case has been known, in which the valve of the cæcum has presented at the annus.

Lecture 35, is on the LIVER, GALL BLADDER, AND GALL STONES. Our author sets out by remarking on the common opinion, that the bile is secreted from the blood of the vena portæ, and seems to concur in the theory; but he has seen a case in which the blood returned from the intestines, went directly to the vena cava, near the emulgent veins, and still, the liver secreted real bile. The hepatic artery was one third larger than usual. Hence it follows, that bile *may* be secreted from *arterial* blood. He seems disposed to advance the opinion, that the secretion of bile, from venous blood, is a mere economical provision. We can scarcely doubt, however, that as it respects the blood of the general system, and especially of the abdominal viscera, the bile is an excrementitious fluid. That it is made to perform a function in the intestinal canal, is no objection to this view of the subject. We think, therefore, that although this fluid has been secreted from arterial blood, its proper source, is that which is returned from the stomach and bowels. This blood we have little doubt, in reality, differs from the other blood of the body, in having matters blended with it by absorption from the stomach, which it is necessary to the welfare of the system, should be eliminated.

Our author's remarks on the composition of the bile, present nothing deserving of particular notice. He is a believer in the contractility of the gall bladder. On the sympathies of the liver, through the instrumentality of the nervous system, he speaks briefly and pertinently; but *all* the injuries to the general system, which hepatic diseases occasion, is not through the medium of the nerves. Retention in the blood, of that which ought to be separated from it in the liver, undoubtedly gives rise to a part of the mischief, and is perhaps a main cause of the depression of spirts, and the distressing nervous irritation which attends hepatic diseases.

From his observations on the morbid anatomy of the liver, we extract the following paragraphs:—

"*Morbid Structure.*—So much with respect to physiology; and next, with regard to morbid structure. Here I get over the ground rather lightly, for there are certain organs in the body, which, if the vessels go into a state of diseased action, they seem to me to produce but one, or scarcely any thing else but one kind of morbid structure; it is an infusion of something into the interstitial parts, in larger or smaller masses, and this we call *tubercles.* The liver is diseased—it is tubercular. There are divers specimens of this sort of disease; sometimes the tubercles are very large, and sometimes they are very small. But what is it that is deposited in the interstices? I am ready to grant, that the newly formed matter may be so newly laid down, as to give a solidity to the whole mass, and that is *scirrhus,* not cancerous scirrhus, but a solid state of the liver. It is, however, generally in masses, so as to constitute tubercles. Now here is a liver [*presenting a large preparation*] where the tubercles are distinctly shown; they are very large tubercles. I am accustomed to introduce this preparation by saying, it is a very small slice off a large liver; for positively the patient from whom it was taken, had the whole of the abdominal cavity filled up with the liver; it went down even into the pelvis. Before her death, some speculated about the nature of this large hard substance. Some thought it arose out of the pelvis, and went up to the hypocondrium; and others thought that it proceeded from the hypochondrium down into the pelvis: I thought with the latter class. But what was astonishing, was, that the patient never had any symptom of a diseased liver. There was no sickness, nor any thing that may be generally observed, as indicating disease of the liver. But you are to understand that these tubercles, to use the language of Mr. Hunter, may be considered rather as disease *in* a part, than *of* a part, for in this state the internal parts of the liver will secrete bile; and it does happen, that very diseased livers will sometimes secrete very good bile; I have known it repeatedly.

Then, with regard to these tubercles, the question is, are they vascular? And that is a question we cannot determine; there are vessels going through them, but whether they are vascular or not, we cannot tell. This relates to the tubercles of the liver and lungs; and in either case, it is notorious that the tubercles *suppurate.* You meet with abscesses there, and those abscesses are, no doubt, suppurated tubercles. I have seen numerous abscesses of this kind, which have discharged half a wash-hand basin full of matter, and yet the patients have recovered and done well, the rest of the liver having been sound; and I have known, as I believe, one tubercle breaking in upon another, abscess after abscess forming, and the matter issuing from the side. I remember a man very well, who was a captain of a ship, and of course, a man who had drunk a great deal of *grog.* He was in that situation, and the disease in the liver communicated with his bowels. Now I have seen this. The abscess has been dressed while the patient was lying in bed. A rumbling has taken place in his bowels, air has got into them, puffed them up, and actually blown off the dressing!"—pp. 135–137.

From the liver, our author ascends to the lungs, and discusses with some earnestness, the question of the irritability of the air cells. As this is an inquiry of interest, and the profession are much divided in their opinions, we shall transcribe a considerable part of his reasonings on the subject:

"*Irritability of the Air Cells.*—With regard to their irritability, these air vessels are primarily irritable; and the question is, whether these vessels, which permeate the lungs, are irritable or not? Now who can tell that? It is believed that all the mucous membranes of the body are irritable. I do not believe that any body can demonstrate an irritable mucous membrane in the urethra, and yet that part has been known to be of considerable irritability. Now it has been a question among surgeons and physiologists, as to the irritability of these vessels in the lungs; and you may ask my opinion on the subject, or I may at once tell you, that I have no doubt of the air vessels of the lungs being irritable. What is to be our guide? Our senses are insufficient to inform us; why then, observation of course must. Ordinarily, respiration may be said to be a mechanical process. We enlarge the capacity of the chest by the *intercostal muscles*, and the air is forced into the lungs. We diminish the chest, and the air is forced out of the lungs, just as if you were using a pair of bellows. Ordinarily, respiration is carried on merely as a mechanical process; but, extraordinarily, do we not find manifestations that air cannot get into the lungs, though we do endeavor to enlarge the chest? You know you never could lift up the board of the bellows, if you were to stop up the holes that admit the air—and why? On account of the immense weight of the atmosphere. Just so, if you were to put a rope round a man's neck, and stop the air from entering into the trachea. It is not the strongest man that ever lived that could afterwards enlarge that man's chest; to do that, would be to lift up an immense load of air. A man having irritable lungs, may be sitting comfortably enough at the fire-side, but a little smoke comes into the room, and he can breathe no more; he gasps for breath—he cannot enlarge the chest, and he finds the utmost difficulty in respiring. But where is the difficulty? Where is the sensation of pain and contraction? Why, in the lungs themselves; the hindrance is there; I believe it is all irritability."—pp. 139–140.

On the following page, he expresses the conviction, that asthmatic breathing is generally dependent on disorder in the digestive organs; in evidence of which, we ourselves have met with many striking cases. Hence, in part, the great palliative influence of emetics in the early stages of asthma. But why does not gastric disease produce pulmonary irritation in all? This can only be attributed to a difference in the vital properties or organization of the lungs, by which in some persons, they are proof against disorders of the stomach, while in others, from an unhealthy disposition, they readily take on sympathetic action. Now emetics exert a decided influence on the pneumatic system, and hence, in part, the benefits which are derived from this class of medicines, in these gastro-pulmonary maladies.

His observations on the physiology of the lungs, embracing their connexion with the heart, and the production of animal heat, are in the main correct; but offering nothing new, we shall pass them by, to speak

Of the Heart. Devoting a couple of pages to its *malformations*, he proceeds to treat of its *morbid anatomy*. In the many cases of extreme dropsy in the pericardium, that he has met with, he has constantly found "a very small and very quick pulse." When treating of the morbid appearances produced by *pericarditis*, he takes occasion to mention the phenomena of that disease, as he has observed them in his own practice; and although in a late number, we have given a history of them from Lænnec, we are tempted to transcribe what our author has seen:—

"*Symptoms.*—Now I have always, in this Lecture, conceived some account of the symptoms which I myself have known in cases of pericarditis, useful to be mentioned. Many a man feels a pain in the heart, when there is no disease there; but I never knew any one who was labouring under an inflammation of the heart who complained of pain in the part actually affected. They either did not complain of pain at all, or referred it to some other part, not in the region of the heart. I have known them complain of pain in the region of the liver. But in those cases of acute pericarditis, it is not to be supposed that the disease which affects the heart will not affect the flesh of the heart, and will not render it irritable. I have known it to be extremely irritable, having the greatest pain, acting with the greatest force, and then, as if exhausted, it has gone on with its function in a very slow manner. Now, in describing this pericarditis, I tell the case of a physician, who had come from the East Indies, and that perhaps might have tended to mislead the minds of his medical advisers, for he died, and they really knew not what the nature of his disease was; but yet it was distinguished by this peculiar symptom that I am describing. He referred his pain to the region of the liver, as if immediately coming from the liver. He was treated as if he suffered from hepatic affection merely; but the most curious circumstance was, that at one time his pulse would beat so quickly as not to be numbered, and that then it would become exceedingly slow. Really, towards the last period of his life, his attendants have gone out of the room, not liking to witness his last agonies, feeling certain, that from the state of his pulse, he could not survive many minutes; and returning into the room again, they have found him in a languid state, with a pulse beating very low indeed, perhaps not more than forty in a minute; just as if the heart was thrown into fits of irritability, in which fits it would act with the utmost vehemence, till it became exhausted, and then carry on its functions, as I have expressed it, in a very languid manner."—pp. 154–155.

His remarks on the morbid anatomy of the muscular substance and valves of the heart, we shall not analyse, as presenting nothing with which the Profession is not familiar; and shall finish what relates to that organ, with an extract on its sympathetic affections:

"*Sympathy.*—With regard to *sympathetic affections*, an organ may be made very irritable and fidgetty from sympathy with another organ, and the sympathetically affected organ may be getting into the worst state of the two. Now I am convinced that disorder of the digestive organs will materially disturb the heart. In short, there are two sets of organs with which I say the heart peculiarly sympathizes; the one is, the *head*, and the other the *alimentary organs*. Of how it is caused by the *cerebral* affection, I have already told you; I am now speaking to you upon the other. But if an organ is kept in a state of irritation, it will go into disease; it will lead to disease—to *organic disease*. These words seem to be objected to by medical men; and then they say, there is *functional* disease which leads to *structural* disease.

Well, then I say, we must endeavor to relieve this functional disease; and that which leads to structural disease in the heart may be liable to lead to rheumatism in other parts. I can only tell you, I have had, all my lifetime, one of the most irritable hearts possible; and sometimes I have thought I should die, just as John Hunter did, of an affection of the heart; but that was, perhaps, only a hypochondriacal feeling. I remember when I was young, that my heart used to beat at such a rate, as to make me think that I had an aneurism. It did not get worse, but it was always worse after dinner. However, by degrees I ceased to think about it, and perhaps it diminished—perhaps it did. I was first reminded of this again, by being excessively distressed and annoyed from attending upon a patient who died. When I laid down in bed of a night, my heart intermitted to such an extent, that I thought it never would beat again. Then it would go on with the utmost vehemence for a time, but as the anxiety of my mind subsided, so this state went off. Then I was next reminded, that my heart might put my stomach out of its order, from my own observations; then my heart was at its vagaries again. I have known it to beat 160 in one minute, and not 60 in the next, intermitting in the strangest way possible. However, my stomach was wrong, and being interested, as you may be inclined to suppose, in this case, I resolved to consult a physician. I let him feel my pulse, and he said, 'You have got a touch of *angina pectoris*.' Well, but, said I, telling me what I have got, is not of so much consequence as telling me what I am to do. He said, 'You are not to take wine—you are not to take this, that, and the other thing,' all which I attended to. I was extremely hypochondriacal. He recommended me to sleep in the country. I did so; and I remember once, just by the friction of my shirt, I had produced a large blister over my heart, and when I was stripped, I found my shirt all over serum and blood. Now this irritability I have known in many other cases, and I felt, personally, that relief from taking the *blue pill*, which I never could have believed could have been produced, had I not felt it. An *Irishman* once said to me, 'Oh! sir, I shall be bound to pray for you as long as I live, and *ever afterwards*. I declare to God, before I took those pills, I was in that state which I should have been thankful to any body who would have come and blown my brains out; but ever since I took them, I have been happy.' "—pp. 161-163.

Leaving this organ, he returns to the pulmonary, and treats of the physiology of the voice, and of the morbid anatomy of the lungs:

"This is one of the organs in which, when diseased action is produced, the disease is tubercular, as in the liver and spleen. I know the matter may not be aggravated, it may be diffused, it may make a sort of solid substance, but it is tubercular. This is what we call a consumption—tubercles in the lungs. Now these tubercles are of various kinds and various sizes; they are of all sizes and diversities of texture, according to the constitution. Then we may question whether these break into the lungs. You have them larger or smaller. Sometimes tubercles are organized or not? We know that they suppurate and a great deal of matter is spit up after the tubercles break; and this is the state of the lungs in those who die consumptive. Certain it is, you do see abscesses in lungs not tubercular."—p. 168.

"Now, with regard to the lungs, what can produce an irritable disease of pulmonary irritation? I am very well convinced, that stomachic irritation will; and I am very well satisfied that this is the primary state of the disease. I know that many gentlemen who have been educated at this hospital, have obtained very considerable credit to themselves, in curing or relieving some consumptive people by putting their stomachs to rights."—p. 169.

That many cases of consumption are of dyspeptic origin, we have no doubt; but many more appear to us to be primary affections of the pulmonary organ, and unsusceptible of being cured by remedies addressed to the stomach and liver, or indeed by any known means.

On the value of morbid anatomy, generally, he holds a sentiment from which we are little inclined to dissent:

"This morbid anatomy, which people dread so much, does not appear to me to be of primary importance in the study of our profession, there is such a great diversity of appearances; but organs of certain construction are liable to certain diseases, and those are easily recognised. The grand thing to be attended to, is, that what produces a state of irritation, and brings on a state of vascular action, will cause those diseases."—p. 169.

The 37th Lecture treats of the Absorbent Vessels and Glands. These vessels are exceedingly similar to veins. They anastomose very much with each other, in which Mr. A. sees a great good, as their contents, drawn from the different tissues of the body, are thus commingled before they reach the blood. He believes, moreover, with Hunter, that these vessels, as well as the other vessels of the body, exert a modifying influence on their contents. The glands are exceedingly vascular, that is, supplied with arteries and veins. It is uncertain whether they are cellular, or composed chiefly of absorbent vessels, rolled upon each other. It was for a long time supposed, that the brain was destitute of these vessels; but the Italian anatomists Morgani and Mascagni assert, that they have injected them in that organ, we believe, however, that they are but few in number. The following pathological remarks seem worthy of being remembered:

"Now, then, the superficial absorbents of the thigh come to the groin, under a number of glands that are situated about Poupart's ligaments. They are generally five in number, and are arranged, as it were, into two rows, one row above the ligament and one row below it. These are the glands in the groin which are so often diseased. There is an *inguinal phalanx*, as they call it, and a *femoral phalanx*, and this is said to deserve the attention of the surgeon, inasmuch as it may denote the cause of disease. If, for instance, you have one of the glands of the inguinal phalanx diseased, you may suppose it arises from irritation in the genitals, in the testicles, and so on; but if it be in the lower row, it may arise from ulcers in the leg. Often where there is tumefaction in the leg do you find disease in this part. Often when my opinion has been asked about a swelling in the groin of a lad, suspected to be bubo, I have found it to be in the lower row, and then I have said immediately, no, it is not a bubo. Upon an examination I have found that the lad had some irritation, or sore about his heel, which had communicated irritation to these glands."—p. 180.

Mr. A. discusses the subject of venous adsorption, and comes to the conclusion, that it does not exist. We must say, however, that his facts and reasonings, do not in our opinion settle this controverted point, or even clear up any of the difficulties that surround it. His attempt to explain away the experiment of Majendie, who killed an animal by introducing poison into a limb, separated from the body except by the columns of blood, will not be regarded as successful. Concerning absorption itself, his opinion that we are ignorant of the mode, will be more generally received.

The next lecture opens with the Kidney and the secretion and composition of Urine, relative to which, he is so far behind Prout and some other writers, that we shall not dwell upon them. In regard to *diabetes mellitus* he is a disciple of Rollo.

From the kidney our author proceeds by a natural anatomical transition to treat of the female *organia genetalia*, to which he devotes forty pages; but the great length to which our analysis has already extended, requires us to omit any notice of these lectures.

In the middle of the 39th, with a most unnatural transition, he proceeds to treat of the Cranium; relative to which we shall lay before our readers a single practical extract:

"Now, why do we learn all the particulars of the different bones of the head by rote? For the practice of our profession, and without such learning we could not practice it. Where may you trephine? Draw a horizontal line three parts above the orbit of the eye, and you may trephine where you list, save and except you may not do it precisely on the middle, because then you would come on the *frontal spine*. If you were to trephine lower down, at the sides, you might penetrate into the orbit of the eye. A person may say, 'Surely I may trephine a little lower than this;' but, I ask, 'Why should you wish it? Are you not almost as low as the bottom of the skull? I say, making general rules, we ought, if we err at all, to err on the side of safety. It is foolish to walk on the very brink of a precipice, if the same good can be effected by keeping at a little distance from it. This, then, is the rule;—*Do not* trephine on the *middle* of the os frontis, for you do not know where this frontal spine, or *crista interna*, is to be found. They say, if the sagittal suture is seen continued down the os frontis, this spine will not be found; but this is not true, for I have seen it in such a scull.

You ought not to trephine over the *arteria meningia media*, if it is possible to avoid it. Neither would a man trephine, so as to open the *longitudinal sinus*, if he could help it. There is such inequality in the bone at this part, that there is great risk in opening it if the trephine is applied here. I know it has been often opened."—p. 239.

"Now, what can you do *surgically* to the *os occipitis?* You may trephine it on either side of the perpendicular ridge; but if you have any thing to do with the under part, you must cut off a man's neck to get to it. I know there are cases on record, where bones have been taken away, and so on, from this part. But I speak generally; and if I err, I give you admonitions on the side of caution."—p. 241.

Dismissing the bones of the head, our author descends in the same lecture, to the PANCREAS, concerning which he presents us with nothing new, except peradventure it be found in the following case:

"I shall now tell you the history of a cancerous pancreas, and as those histories are not very commonly recorded, I have some satisfaction in doing this, inasmuch as it seems to be elucidating the physiology of the pancreas, and, in a degree, showing that its *function* is not of very grand importance. There was a medical man in London, a most respectable man, a man of considerable knowledge and judgment in his profession, but especially in the *pharmaceutic* department of medicine. He was, in short, a very old, and a very respectable apothecary, known, of course, to all the physicians in this town; and they were at one time very much, I may say, dependant upon such practitioners as I have described, and paid great court to them. I mean to tell you that this man was attended by the best physicians and best physiologists in London, but not a soul got at the real nature of his malady. It was not ascertained till he was dead. It was observed that he took aperient medicines out of his own shop at the commencement of his illness. His bowels, therefore, seemed to be in some degree constipated; but that was not wonderful. At the commencement of his illness, he looked ill—I mean like a person that had some serious disease within him. Well, I never saw any one who was in that state, but where it was betrayed in his physiognomy. This is a common observation. I say to patients every day, 'Oh! you will not die yet—you do not look as if you were going to take your departure.' This is a common observation. We go along the streets, and seeing some poor fellow pass us, we say, 'Ah! poor fellow, he has not long to live in this world, I am sure.' However, the individual I allude to, looked as if he had some important disease within him. Nevertheless he ate his food, and I suppose he kept his bowels regular. When the pancreas began to ulcerate, it produced no effect but that of local pain. He complained of pain in the pit of his stomach, which gradually extended in its dimensions; but what was the cause of that pain no one could find out. As it advanced, he was constantly stooping forwards. Towards the latter part of his life he lay in bed, propped up with pillows, to lessen the pressure that might have been made on the pancreas. He still had an appetite, and his bowels were kept regular. He found that when he ate more than he should have done, that that aggravated his pain; so much so, that he learned to relieve himself by poking his finger down his throat, and pushing forward some of the food through the stomach. [His fingers were certainly of an enviable length.] The pressure of the food on the pancreas increased the pain. He also found that a *clyster* relieved him, probably by unloading the *colon*, and lessening the pressure that might previously have taken place on the pancreas. Thus he went on, gradually becoming worse and worse till he died, and then his pancreas was found to be in a horrible state of ulceration from end to end."—pp. 243–245.

Next succeeds the SPLEEN, on the functions of which we shall make an extract:

"*Physiology.*—Now for the physiology of the spleen; and it was known in ancient times that if a man was wounded in the wars, and had his spleen slip out of his belly, it came away, perished, and yet the man did well. Well, upon the revival of philosophical inquiry, Malpighi took away the spleen of a dog, and he has given a history of the proceeding. According to his account, the dog has rather benefitted by the operation than otherwise, or the dog had more appetite, and became more salacious than he was before the operation. When Malpighi had led the way, every person who could get a dog, was cutting out the spleen and publishing the history of the case. Haller has made a general review of the histories of those *unsplenic* dogs, and the summary is just what we would naturally expect it to be; namely, that though some of the dogs did well for a time, that they became ill, pined away, and died. This is just what we should naturally expect, because no one could suppose that the spleen was made in vain and for no purpose. Haller thought that they died, because they had not got a preparation of bile; for in Haller's time it was thought that all the abdominal viscera were but organs for the preparation of bile—that they wrought a change in the blood, that fitted it for the secretion of bile; and every anatomist has observed that the blood of the splenic vein does not coagulate like the blood of other veins. This is an observation which should be attended to, for we really do not understand the physiology of the spleen. Then Haller, you know, had a theory to support; and, therefore, probably he might consider this as a stronger proof than he otherwise would have done. But the opinion that bile could alone be prepared from venous blood and from the *vena portæ* which contained blood of a peculiar quality, is now, I consider, refuted. Then, why should so large an artery go to the spleen, to have the blood simply to pass through it, without preparing any thing, and to return by the splenic vein? The only rational explanation that has been given of this, is, that if the circulation through the spleen can at any time be impeded, it must throw an abundance of blood into the other branches of the *cœlic artery*, so that there would be a most inordinate quantity of blood poured upon the stomach and the duodenum at the time digestion was taking place. It is supposed that the pressure of the great end of the stomach upon the spleen must impede the blood through the spleen, and cause it to flow in abundance in those parts where it is most wanted. I say this is the only rational explanation that has been given of it, and also the only solution of the problem of the unsplenic dogs: for if the spleen could be taken out and the wound healed up without injury, then there would be an inordinate quantity of blood thrown to the stomach and other parts; so that Malpighi's dogs might well have a better appetite and be more salacious than before they took leave of their spleen. I say this is the only rational theory, but still it may not be true for all that; and there is one staggering fact which I hear of, which is, that the spleen seems to be largest when the stomach is full. That is a thing I never examined for myself, and I can tell you no more about it. Dr. Haighton took a great deal of pains with this theory, and examined the blood of the splenic vein. He did not find it different from other blood. But he seems to me to have left off where he should have begun. It was his business to have shown that a solid pressure upon the spongy part would impede the circulation; and then to have shown that the stomach was adequate to produce that pressure, which he did not show."—pp. 246–248.

On the subject of the Blood Vessels and Blood, our author adheres so closely to his great master, John Hunter, whose work on the same subjects, is, or ought to be, in the hands of every physician, that we shall limit our excerpts to the closing paragraph:

"Now that is all I have to say on the subject, but I must add, that it is a very satisfactory examination, as it shows you that the blood contains the rudiments of all the body. What do you find that the body is composed of, but of *fibres*, some insoluble, and some soluble? All this is to be found in the blood. You have also something in the blood which is susceptible of considerable mutations of color, and you see such mutations take place throughout all the body. I say, we know but little, but that there is a sort of satisfactory conclusion come to from what we do know."—p. 261.

To the vessels and blood, succeed the Muscles and Nerves. What our author says on the structure and functions of these parts, is not new; nor has he by any means embraced all the known facts. He does not speak of the curious microscopic observations of Dr. Edwards, on the relations between the ultimate extremities of the nerves and the muscular fibres, nor make a single allusion to Mr. Bell's discovery of a system of respiratory nerves, the most interesting of all the modern contributions to the physiology of the nervous system. We have already said, that our author is a distinguished opponent of the doctrine, maintained by Mr. Lawrence and others, that the phenomena of life, spring from organization alone. In the following extract, his ideas are set forth:

"I say the physiology of the nervous system, the physiology of secretion, the physiology of muscular action—all tend to convince my mind that Mr. Hunter's notions of life are true, and that there is some subtile material invisibly commixed with the visible fabric of the body, operating on each part, so as to produce the phenomena of the vital functions.

The deduction that I draw from the whole of the productions of modern anatomy, is a very curious and a very simple one. We know no more, after all, than what common sense dictates. We know no more than what a man of sense, in the first ages, would have believed, upon considering the phenomena of life, which is, that there is living in beings an organized structure invisible in the body; and in the common material a principle of life pervading every part—a sentient and rational faculty connected with the brain, but each apparently distinct from the other, though they are all intimately connected, and operate one with the other. Here I go a step beyond John Hunter; I believe sensation to be as distinct from life, as I do life to be distinct from the materials of our body. I say, that is a step beyond John Hunter; but it is an opinion that the most intelligent of mankind have always entertained; it is no new opinion. Have you not life without sensation? Cannot I ask that question? Do you believe that *vegetables* feel? There are some people who have a propensity to take things up by the wrong ends; and I say, if they had a poker presented to them with one of its ends *red hot*, they would lay hold of the red hot part rather than the other end, and burn their fingers of course. Dr. Darwin has done this; he begins to certify things, deducing inferences from the conduct of man. He says, man performs certain actions, because he *reasons;* therefore he infers, that *brutes* do the same thing, because they reason. This is common belief. Now I really do not know how people can believe it. I know not what kind of faith they possess, I am sure; but I do not believe that *instinct* is *reason.* I believe that it is *a blind impulse*, inducing actions far superior, in their effect, to what reason does; but still it is a blind impulse."—pp. 267–268.

His views of sensation and intellection are stated in a succeeding page, which we shall also transcribe:

"Well, I say, can you believe that life may exist without reason? I do believe it. I do not believe that vegetables feel, or that the lower order of animals feel in the degree in which we do; and as far as we know, all feeling is placed in one point in animals, which is a little *tubercle* at the top of the medulla spinalis. As soon as this tubercle begins to form, so soon we begin to find something sentient; and as this knob begins to grow, and becomes complex, so do animals have additional faculties and powers.

Now that is all I have to say upon the subject; for how any man, or set of men, can possibly believe that those faculties are the result of mere replications of the brain—for such the convolutions are—is what I cannot understand. I have no faith of that kind. The brain must be allowed to be the organ by which a diversity of perception is prone to act. We have divers perceptions, and divers faculties; but it is said that those are only the simple replications of the brain, which form the convolutions. Well, that is very strange. And how is it argued? Why, we have organs by which we see and hear, and smell, and taste—giving us diversity of feelings. Now, the eye does not see. The reason of the organization of the eye, you understand perfectly well, is to transmit the rays of light arranged in the same manner as they present themselves from the different visible bodies. The organization of the ear you understand. But the eye does not see, nor the ear hear. The notions we form must be dependent upon the peculiar properties of that which is perceptive, and it is wonderful; for what can we make of it, if we consider it intently? What is there around us, but matter in different states of motion and of rest? As subtile matter is reflected from objects, it is arranged on the eye in the same order in which it was produced from the surrounding objects. Well, there is the magnetism of the eye. Vibrations take place, and the agitation of the fluids in the labyrinth of the ear produces the sound. Oh! but it comes to this, that the vital actions of the nerves take place, and those are propagated to the brain; hence are the different sensations produced. All you can make of it is, a sort of vibratory motion; and how from one set of vibrations we can see, and from another we can smell, and from a third we can hear, is certainly very curious; but they are all vibrations, or tremors of the nerves. I say all this I can only attribute to the very curious properties of that which is perceptive, and that that has divers faculties superadded, capable of being acted upon by the brain, a subject which no one can consider without wonder. But I cannot believe that the faculties are occasioned by the magnetism of the brain. Now here the subject becomes *metaphysical*. I do not want to enter into metaphysics in this course of Lectures, but I believe if we were to go into metaphysics you would be of opinion as I am, that there is a unity of all the senses with the brain. They put the organs all in different places; whereas, I ask, how can you account for it? Many of the actions which are performed are the effects of compound motives. There is no answer that can be given, but that it must be done by sub-committees of the different organs—by the board of control. Now, I say, if you go to the board of control I am contented. However, every opinion has its day. But the opinions which I am in the habit of teaching here have endured from the first ages to the present time; therefore I think they will stand on. I think that all wonders—that all novelties of the day will decline, but that those opinions will flourish?"—pp. 269–270.

On the subject of SYMPATHY, Mr. A. is a follower of his celebrated countryman Whytt, whom, however, he never mentions. We are indeed, not a little astonished, that the name of this able sympathist is not oftener pronounced by those who profess to explain most of the morbid phenomena of living bodies by sympathy. His treatise on sympathetic affections, and on the agency of the nervous system, in producing them, has always seemed to us a masterpiece of physiological pathology. One of his favourite propositions was, that it is by the association of nerves with each other, at their central extremities, that sympathies are established; and this proposition has received great support from the discoveries and reasonings of Mr. Bell, on the specific character and non-communication of the different nerves. But our limits do not permit an exposition of this interesting subject, and we shall dismiss it with a short extract:

"*Sympathy of Nerves.*—The dissection of the nerves sometimes has rendered it reasonable to suppose, what we are justified in supposing from every other part, that all parts of the body sympathize with one another. They show us the cause of sympathetic affections in divers diseases, and throw light on other subjects. It is, however, yet an indistinct light, but as the sun has risen, I hope it will go on and illuminate the subject more completely."—p. 288.

Disposing of the nervous system, our author proceeds to treat of the peculiarities of the fœtus, and with them finishes the anatomical part of his course. The remaining lectures, which extend through more than eighty pages, are on Operative Surgery, and contain many important rules and principles; but as the limits of our present number, although extended beyond the regular distance, will not admit of a suitable analysis of these practical lectures, we shall reserve them for some future occasion; and bring our review to a close, with a few general observations, on the plan and style of what we have travelled over.

*Method* says Linnæus *is the soul of science;*—an apothegm which should be inscribed on the portfolio of every public teacher. That our author has seldom had it before his eyes, will be seen and felt by all who undertake the perusal of his lectures. His subjects do not, in general, succeed each other in a natural order; and the subordinate parts of the same topic are often transposed in a manner, that could not fail to bring upon a writer of inferior genius, the imputation of deficient perspicacity: In Mr. A. it will of course pass for negligence. We cannot but marvel, however, that in the long period through which his prælections were delivered, he should not have been, instinctively, impelled to infuse into them the charm of a more natural arrangement. To himself it would have been a convenience: to his pupils and readers an unquestionable advantage. On the score of originality their claims are not very great, if we look beyond the cant phraseology in which they are dressed up. They are rather eccentric than original. But it is the province of a public teacher, to exhibit that which is already known, rather than to make new discoveries. Mr. A. nevertheless has taken and will support, an elevated rank among the original writers of the age; but it is on his previous writings that he must rely. In his lectures, however, we everywhere find indications of a powerful and perspicacious mind:—proud, whimsical and obstinate;—prone to do good, but impatient of interference in the manner—benevolent but rude.

Of his independence in thought and action, many anecdotes have been related, some of which do credit to the profession—while others present it in a dubious attitude. With such qualities, it is not remarkable that Mr. A. maintains an extended influence in the British metropolis. But we would warn those who aspire to a similar influence, elsewhere, not to imitate the oddities and ill manners of Mr. A. unless they have good assurance, that nature has endowed them with his genius, industry and perseverance. Without these, violations of decorum bring no reward; and with them, we may add, such violations are unnecessary.

But his style is in many respects still more objectionable, than the neglect of method, of which we have spoken. We are aware of the great influence of a *droll* and *gossiping* style, upon the attention of an audience, who are, day after day for months, to listen to the same professor. But we humbly conceive, that its efficacy may be greatly diminished, by its unceasing administration. Much of our author's drollery moreover, is coarse and unattractive, not to say offensive. A colloquial style it is true, is not without its advantages, but they are felt by auditors rather than readers. Many things seem well enough in the lecture room, that appear very different in the Library. Printed lectures, to be read with permanent interest, must be composed with some degree of dignity; and the neglect of this on the part of distinguished men in the profession, must of necessity contribute to vitiate the taste of its inferior members. We cannot but regret, therefore, that Mr. A. had not to a certain degree revised his lectures; and conformed them to the models which he, himself, had previously laid before the profession, in the compositions which he deliberately prepared for publication.

## Figures and Tables

**Figure f1:**